# 

**Published:** 1909-07

**Authors:** 


### BOOKS RECEIVED.

The Practical Medicine Series. Ten volumes. Issued under the general editorial charge of Gustavus P. Head, M.D., Professor of Laryngology and Rhinology in the Chicago Post-Graduate Medical School. Volume III. Eye, ear, nose and throat. Edited by Casey A. Wood, Albert H. Andrews, Gustavus P. Head. Series 1909. Chicago: The Year Book Publishers. (Price, $1.25; entire series, $10.00.)

Saunders's Question Compends. Essentials of Medical Chemistry. By Lawrence Wolff. Seventh edition, revised by A. Ferree Witmer. Philadelphia and London: W. B. Saunders Co. (Price, $1.00)

American Pocket Dictionary. Edited by W. A. Newman Dorland, Assistant Obstetrician to University Pennsylvania Hospital. Sixth edition. Philadelphia and London: W. B. Saunders Co. (Flexible leather, $1.25.)

Progressive Medicine. A Quarterly Digest of Advances, Discoveries and Improvements in the Medical and Surgical Sciences. Edited by Hobart Amory Hare, M.D., Professor of Therapeutics and Materia Medica in the Jefferson Medical College of Philadelphia. Vol. XI. No. 2, June, 1909. Octavo, 317 pages, with 52 illustrations. Per annum, in four paper-bound volumes. Lea & Febiger, Publishers, Philadelphia and New York. ($6.00 in cloth, $9.00, net prices.)

Report of the Commissioner of Education for the year ended June 30. 1908. Vol. 2. Washington: Government Printing Office.

Handbook of Diseases of the Rectum. By Louis J. Hirschman, lecturer on rectal surgery, Detroit College of Medicine. Octavo, pp. 374. St. Louis: C. V. Mosby Medical Publishing Co.

Dietetics for Nurses. By Julius Friedenwald, Professor of Gastroenterology in the College of Physicians and Surgeons, Baltimore. And John Ruhrah, Professor of Diseases of Children in the College of Physicians and Surgeons, Baltimore. Second edition. 12mo. pp. 395. Philadelphia and London: W. B. Saunders Co. (Cloth, $1.50.)

Modern Medicine. Its Theory and Practice. In Original Contributions by American and Foreign Authors. Edited by William Osler, M.D., Regius Professor of Medicine in Oxford University, England. Assisted by Thomas McCrea, M.D., Associate Professor of Medicine and Clinical Therapeutics in Johns Hopkins University. In seven octavo volumes, illustrated. Volume VI, Diseases of the Urinary System, of the Ductless Glands, of the Muscles, Diseases of Obscure Causation, Vasomotor and Trophic Disorders, Medical Aspects of Life Insurance. Lea & Febiger, Publishers, Philadelphia and New York, 1909. (Price per volume: cloth, $6.00; leather, $7.00; half morocco, $7.50 net prices.)

Vaccine and Serum Therapy. By Edwin Henry Schorer, Assistant Professor of Parasitology and Hygiene, University of Missouri. Octavo, pp. 131, illustrated. St. Louis: C. V. Mosby Co. (Price, $200)

Physiological and Medical Observations among the Indians of Southwestern United States and Northern Mexico. By Alex. Hrdlicka. Washington: Government Printing Office. 1908.

Diet in Health and Disease. By Julius Friedenwald, Professor of Gastroenterology in the College of Physicians and Surgeons, Baltimore, and John Ruhrah, M.D., Professor of Diseases of Children in the College of Physicians and Surgeons, Baltimore. Third edition. Octavo, pp. 765. Philadelphia and London: W. B. Saunders Co. (Price, $4.00.)

Myomata of the Uterus. By Howard A. Kelly, Professor of Gynecology in the Johns Hopkins University. And Thomas S. Cullen, Associate Professor of Gynecology, Johns Hopkins University. Octavo, pp. 723. Illustrated. Philadelphia and London: W. B. Saunders Co. (Price, $7.50.)

International Clinics. A Quarterly of Illustrated Clinical Lectures and especially prepared articles on Treatment, Medicine, Surgery, Neurology, Pediatrics, Obstetrics, Gynecology, Orthopedics, Pathology, Dermatology, Opnthalmology, Otology, Rhinology, Laryngology, Hygiene and other topics of interest. Edited by W. T. Longcope, M.D., Volume II. Nineteenth series. Philadelphia and London: J. B. Lippincott Co. 1909. (Cloth, $2.00.)

Treatment of the Diseases of Children. By Charles Gilmore Kerley, Professor of Diseases of Children in the New York Polyclinic Medical School and Hospital. Second edition. Octavo, pp. 629. Philadelphia and London: W. B. Saunders Co. 1909. (Cloth, $5.00.)

Hydrotherapy. By William H. Dieffenbach, Professor of Hydrotherapy, New York Homeopathic Medical College and Flower Hospital. Octavo, pp. 267. Illustrated. New York: Rebman Co. (Cloth, $3.00.)

Treves's Operative Surgery. New (3d) Edition. A Manual of Operative Surgery. By Sir Frederick Treves, Bart., G.C.V.O., C.B., LL.D., F.R.C.S., Serjeant-Surgeon to H. M. the King, Surgeon-in-Ordinary to H. R. H. the Prince of Wales, Consulting Surgeon to the London Hospital; and Jonathan Hutchinson, F.R.C.S., Surgeon to the London Hospital. New (3d) Edition, revised and rewritten. In two octavo volumes. Volume I, 775 pages, with 193 engravings and 17 full-page plates. Lea & Febiger, Publishers, Philadelphia and New York, 1909. Half-morocco, $6.50, net.

### MISCELLANY.

### INDEX TO VOL. LXIV.

### AUGUST, 1908—JULY, 1909.

Page

ACADEMY of Sciences at Vienna .................. 510
Achievement, a notable ........... 570
Adams: Painting at exhibition in Kunstlerhaus at Vienna ......... 623
Adulteration of food .............. 327
American fleet at Auckland ....... 259
Anesthesia ...................... 243
 lumbar, results of in gynecological operations 323
 moral ................. 274
Anesthetic new, reported by American Consul at Bucharest ........ 682
Anniversary, Roswell Park's ...... 340
Antituberculosis problem, public health in relation to ............ 203
Antivivisection bills, opposition to. . 622
Anusol suppositories in hemorrhoids, etc. ............ 395
 for hemorrhoids ......... 445
Apollinaris ...................... 345
Arhovin Antigonorrheic efficacy of 395
Army Medical Reserve Corps ..... 224
Arsacetin, employment of, in syphilis ........................... 536
Arthritis gonorrheal, antigonococcic serum in ...................... 434
Asthma, pyrenol in pertussis and... 103

BACTERIURIA, internal medication in ...................... 45
Berlin Eye Hospital .............. 510
Blastomycosis in New York State. . 603
Blood pressure, high currents upon. 600
Bone, endothelioma of, following injury ......................... 480

**Books and Authors:**

Abrams: The blues (splanchnic neurasthenia) ............. 59
Adami: Principles of pathology 409
Aikens: Primary studies for nurses ..................... 636
Allen: Vaccine therapy and opsonic method of treatment. 464
American Medical Association: New and nonofficial remedies 638
Ballenger: Diseases of nose, throat and ear .............. 176
Bandler: Medical gynecology .. 61
Bandler: Medical gynecology . 117
Beck: Reference handbook for nurses ..................... 288
Bell: Changing values of English speech ................. 579
Benedict: Golden rules in dietetics ...................... 581
Bickman: Textbook of operative surgery ................ 284
Bonney: Pulmonary tuberculosis ....................... 233
Brickner: Seven hundred surgical suggestions ............. 640
Browne-Crichton, Sir James: Parcimony in nutrition ..... 580
Brunton: Therapeutics of the circulation ................. 462
Bryant and Buck: American practice of surgery ......... 459
Butler: Textbook of materia medica, pharmacology and therapeutics ................ 636
Cabot: Clinical diagnosis and treatment of disorders of the bladder with technic of cystoscopy ..................... 640
Camac: Epoch-making contributions to medicine, surgery and allied sciences .......... 634
Campbell: Textbook of surgical anatomy .................... 59
Casper and Bonney: Genitourinary diseases ............ 520
Chapin: Theory and practice of infant feeding .............. 639
Claiborne: Cataract extraction. 289
Coakley: Manual of diseases of nose and throat .......... 286
Combe: Intestinal autointoxication ...................... 459
Cullen: Adenomyoma of the uterus ...................... 283
DaCosta: Physical diagnosis. . 457
Dana: Textbook of nervous diseases and psychiatry ..... 285
Davis: Consumption .......... 286
Davis: Obstetric and gynecologic nursing ............... 463
Dearborn: Textbook of physiology ..................... 408
DeLee: Obstetrics for nurses. . 460
de Schweinitz: Pulsating exophthalmos ................. 289
Dieudonne and Boldman: Bacterial food poisoning ....... 635
Dudley: Principles and practice of gynecology .............. 282
Findley: Gonorrhea in women. 464
Fischer: Diseases of infancy and childhood .............. 350
Gant: Constipation and intestinal obstruction ............ 519
Goepp: State Board questions and answers ................ 234

Page
Gould: Concerning Lafcadio Hearn ..................... 576
Gould: Borderland studies .... 581
Gould: Righthandedness and lefthandedness ............. 582
Gray: Anatomy, descriptive and surgical .................... 403
Gulick and Ayers: Medical inspection of schools ......... 520
Haab and deSchweinitz I. Atlas and epitome of external diseases of the eye ........... 637
Haab and deSchweinitz: II. Atlas and epitome of ophthalmoscopy and ophthalmoscopic diagnosis .................. 637
Hare: National standard dispensatory .................. 522
Hare: Progressive medicine.... 61, 283, 402, 573
Head: Practical medicine series 234
Herbert: Cataract extraction.. 466
Holland: Textbook of medical chemistry and toxicology.... 633
Howe: Muscles of the eye .... 281
Hubbell: Development of ophthalmology in America ..... 118
Jackson: Handbook of diseases of the skin ................ 288
Jacobs: Campaign against tuberculosis in United States... 465
Jordan: Textbook of general bacteriology ................ 462
Keen: Surgery .............. 458
Kelly: Gynecology and abdominal surgery ................ 406
Kerley: Treatment of diseases of children ................. 460
Kerr: Operative midwifery ... 407
Kerr: The baby .............. 461
Kolle: Subcutaneous hydrocarbon protheses .............. 291
Kopetsky: Surgery of the ear. 457
Lea & Febiger: The practitioners' visiting list ............ 285
Lexer: General surgery ....... 402
Lindsay & Blakiston: Physician's visiting list .......... 290
Longcope: International Clinics, 18th Series, Vol. IV......... 289, 408, 573, 461
McCrea: Modern medicine .... 404
Mallory and Wright: Pathological technic .............. 408
Marshall: Sexual question .... 176
Massey: Conservative gynecology and electro therapeutics ....................... 686
Mellin's Food Co.: Method of percentage feeding .......... 60
Miller: Cure of rupture by paraffin injections ........... 287
Moure: Elementary practical treatise on diseases of the larynx and pharynx ........ 685

Page
Mumford: Surgical memoirs and other essays ........... 405
Munro: Handbook of suggestive therapeutics, etc. ....... 465
Neef: Practical points in anesthesia ..................... 465
Nicoladoni: Anatomie und mechanismus der skoliose.... 638
Ogilvy: Orthopedic surgery ... 574
Osler: Modern medicine ...... 116
Park: Pathogenic microörganisms ....................... 405
Parker: Refraction of the eye.. 287
Pattee: Practical dietetics with reference to diet in disease... 635
Penrose: Diseases of women .. 463
Phillips: Spectacles and eyeglasses ..................... 410
Potts: Nervous and mental diseases ...................... 59
Powell: Pocket Medical Formulary ...................... 522
Ruhrah: Diseases of infants and children ................ 61
Schamberg: Diseases of the skin and the eruptive fevers.. 686
Schmidt: Pain ................ 287
Strong: High-frequency currents ...................... 407
Thorington: Refraction and how to refract .............. 639
Todd: Manual of clinical diagnosis ...................... 288
Upson: Insomnia and nerve strain ...................... 291
von Neusser: Bradycardia and tachycardia ................ 60
Warfield: Arteriosclerosis ..... 466
Watson: Diseases and surgery of genitourinary system ..... 348
Weber: Means of the prolongation of life ................ 457
Wile: Blood examination in surgical diagnosis ........... 635
Williams: Natural history of cancer ................ 61, 291
Wood & Co.: Medical Record visiting list ................ 349
Woolsey: Applied surgical anatomy .................. 464
Ziegler: General pathology .... 405

**Transactions:**

American Medico-Psychological Association, 1907 ........ 521
American Otological Society, 1908 ........................ 523
American Laryngological Association, 1908 ............... 523
American Surgical Association, Vol. XXVI, 1908 ........... 575
American Laryngological, Rhinological and Otological Society ........................ 582

Page
Section on Obstetrics and Diseases of Women of the American Medical Association..... 575
Sixth annual conference state and territorial health officers with the United States public health and marine hospital service .................... 638
Oregon State Medical Association, 1908 ................ 524

**Reports:**

Commission of 1908 to investigate condition of blind in State of New York ......... 521
Commissioner of Education of United States .............. 583
Henry Phipps Institute, 1908... 524

Books received: 61, 118, 177, 234, 292, 350, 410, 467, 525, 583, 641, 687
Bowel and stomach disturbances, overlooked causes for ........... 142
Breech Cases, hints on management of ............................ 129
Brovalol, a new valeric ........... 331
Brush, Edward N.; anniversary dinner ............................ 396
Buffalo Academy of Medicine, activities and interests of 83
General Hospital ......... 448
day camp for tuberculosis opened ........................ 681
Bull and McCosh, honoring ....... 507
Burke: pneumopericardium, report of case ........................ 531

CALOX, oxygen tooth powder.53, 344
Caravia, Eugene: Cuguillere's serum in tuberculosis ........... 156
Cardiac, unusual murmurs ........ 106
Cerebral diplegias, prolonged and tedious labors and forceps deliveries as causes of epilepsy, idiocy and ............................ 353
Cervix, artificial dilatation of ...... 247
Chappaqua Mountain institute, abolition of ........................ 510
Chicago Health Department, pure milk crusade of ......... 450
tuberculosis institute, educational campaign of ... 510
Health Department: Danger of flies ..................... 680
Children, care of ................. 91
chronic constipation in .. 102
school, diseases of ...273, 274
Citizen doctor .................... 477
Clinic, Buffalo Hospital Sisters of Charity ......................... 555
Clegg, Moses: Success in cultivating leprosy bacillus ............. 623
Cocaine, prohibitory duty to be placed on imported.............. 682

Page
Collargol enemata in septic affections ..................... 155
various uses of ........ 495
to children ............. 496
in joint rheumatism .... 496
in cystitis ............... 497

**College and Hospital Notes:**

Buffalo General Hospital ...... 632
Caroline Rest ................... 631
College of Medicine, Syracuse University .................. 58
Columbia University, New York 633
German Hospital, Buffalo, new free dispensary ............. 175
German Hospital, changes in staff ......................... 232
German Deaconess's Hospital, changes in staff ............. 232
German Hospital, nurse's home to be built ................. 516
Harrington Hospital for children .......................... 684
Homeopathic Hospital, new ... 232
Hospital for Contagious Diseases, resolution ............ 280
Howe Eye Hospital, filed incorporation papers ............. 517
Japanese Hospital at Seoul, Corea ..................... 232
Jewish Maternity Hospital, dedication of .............. 517
Kenerson's Dr., cottage, Buffalo, N. Y. ................. 456
LeRoy, prospects for hospital at 684
Medical Department, Western Reserve University ......... 455
McGill University celebrates... 518
Neurological Institute, New York ...................... 632
New York City Hospital....... 632
Providence Retreat, Buffalo ... 455
Tulane University, Dr. Dock's lectures at ................. 280
University of Buffalo, Medical Department .............116, 573
Waldorf-Astoria, first New York Hotel to recognise importance of own hospital..... 517

**Correspondence:**

Animal experimentation, prevention of abuse in—Van Fleet ...................... 338
Food, benzoates—E. E. Smith.. 504
Medical research, council on defense of—W. B. Cannon..... 339
New York Association for the Blind ..................... 447
Researches concerning compensatory diarrhea—George N. Jack ...................... 48
Wistar Institute of anatomy and biology ................ 503

Page
Cottis: Endothelioma of bone following injury .................. 480
Cough, whooping, treatment of .... 95
Cremation in Germany in 1908 .... 509
Cup, individual paper drinking..... 623
Cystitis, treatment of ............ 101
prevention of ........... 105
Cystoscopes, originality and priority in modern .................. 1

DARLINGTON, THOMAS: Individual paper drinking cup .. 623
Deaver, John B.: Banquet in honor of .............................. 624
Diagnosis under artificial light..... 126
of pancreatic affections . 473
Digitalis, infusions of, in heart cases ........................... 330
Doctor on the same plane with a dog, why is .................... 622
Dowd: Prostatic lobes, presentation of specimens ............... 666
Drug, adulteration and food, medical and legal significance.. 132
store, imprisonment for substitution in .............. 276

EBSTEIN: Experiences with medinal-Schering ............ 494
Eclampsia puerperal, renal decapsulation for ................... 305
Education, medical, conference on.. 566
Egg, poisoning by ................ 491
Ellis, Richard: unusual cardiac murmurs ....................... 106
Endometritis puerperal, treatment of 331
Enemata collargol, in septic affections ......................... 155
Epilepsy, idiocy and cerebral diplegias, prolonged and tedious labors and forceps deliveries compared as causes of .............. 353
Erysipelas, case of ................ 667
Eulenburg: Pyrenol in pertussis and asthma ....................... 103
Exfoliativa dermatitis, case of ..... 661
Extrauterine pregnancy, experiences in .......................... 237
Exodin ......................... 102

FALLIERES'S President, first list of decorations ............ 449
Febrile affections, urotropin in lumbago of ....................... 154
Fever yellow mosquito, submission to bite of ...................... 624
Fetterolf, George: symptomatology of tuberculosis of larynx......... 435
Fever, typhoid, new dietetic and injection method of treating ...... 222
Filippino physicians ............... 509
Flies, danger of .................. 680

Page
Food and Drug Adulteration: medical and legal significance ..................... 132
,adulteration of .............. 327
Foster, Frank P.: medical reserve corps ........................... 273

GONOCOCCIC urethritis, acute 546
Gonorrhea, treatment of acute 121
Gonorrheal arthritis treated with antigonoccic serum ............ 434
Grawitz: Scarlet fever, treatment of 495
Greater University of Buffalo grows apace ......................... 570
Greef: Berlin eye hospital, director of ............................ 510
Gould, George M.: Exodin ....... 102
From the patient's point of view ........ 469
Gynecological disorders, rest an important factor .... 146
operations, lumbar anesthesia in ..... 323

HARDY: Double vagina ........ 556
Hayd, Charles Gordon: A notable achievement ........ 570
Health, influence of smoke on ..... 271
department preparing to enforce antispitting ordinance .................. 570
Heart cases, infusions of digitalis in 330
Hemorrhoids ..................... 102
anusol suppositories in ............395, 445
Hepatic derangement, treatment of. 264
Hexamethylenamin (Urotropin) excretion of, in the bile and pancreatic juice .................... 44
Hichens: Messina earthquake, described by ....................... 526
Higher Education Association, announcement of ................. 680
Hip-joint disease incipient, operation for cure of ............... 594
Honors to Dr. Henry Reed Hopkins 569
Hospitals, municipal contagious disease ............... 195
for contagious diseases, need for ............ 450
Howe: Dermatitis exfoliativa ..... 661
Hughes, Governor: Signing of Senator Davis's bill ............ 625
Hughes, Governor: Action of in vetoing C. F. Brown bill ........ 680
Hygiene, industrial and personal... 107
Hypertension, nitrites in .......... 606

IDIOCY and cerebral diplegias, prolonged and tedious labors and forceps deliveries as causes of epilepsy ...................... 353

Page

"If London were painted green," by a leading eye specialist ....... 450

**Illustrations:**

Burke: Appearance of pancreatic tumor .................. 475
Burke: Pneumopericardium ... 531
Fig. 1, percussion dulness, lying on back ......... 532
Fig. 2, percussion dulness, sitting up .......... 532
Fig. 3, percussion dulness, lying on right side ... 533
Fig. 4, showing area of precordial bulging and tympany ............ 535
Conklin: D'Arsonval Cage..... 601
Hardy: Double vagina ........ 557
Howe: Dermatitis Exfoliativa.. 662
Fig. 1, dermatitis exfoliativa 662
Fig. 2, dermatitis exfoliativa 663
Fig. 3, dermatitis exfoliativa 664
Fig. 4, dermatitis exfoliativa 665
Lewis, cystoscopes ............ 2
Fig. 1, Brenner ............ 2
Fig. 2, Boisseau du Rocher (1889) .............. 3
Fig. 3, Boisseau du Rocher (1898) .............. 4
Fig. 4, Guterbock (1895) ... 5
Fig. 5, Fenwick ............ 5
Fig. 6, Albarran (1897) ..... 6
Fig. 7, Nitze, evacuation cystoscope (1897) ...... 7
Fig. 8, Koch-Preston-1898 (air) ............... 8
Fig. 9, Schlagintweit catheter cystoscope (1899) ... 9
Fig. 10, Tilden-Brown-1899 .. 11
Fig. 11, Long (1899) ........ 11
Fig. 12. Kollmann-1900 ...... 12
Fig. 13. Bransford Lewis Air Cystoscope ........... 13
Fig. 14. Wossidlo-1900 ....... 14
Fig. 15. Tilden-Brown-1901 .. 15
Fig. 16. Bransford Lewis universal cystoscope-1906 16
1906 ................ 16
Fig. 17. Nitze-Dittel-1887 .... 17
Mann: Compact ether-freezing microtome .................. 152

Intestinal obstruction ............ 65

**Items:**

Antikamnia Chemical Co; construction of new building ... 120
Antikamnia Calendar. 1909 .... 352
Battle & Co.. dislocations..120, 468
Breitenbach Co. M. J.: Physician's daily memorandum.... 352

JEANBRAU: Collargol in cystitis ....................... 497

Page

KEINER: Croupus pneumonia, pyrenol in ................... 103
Kelly, H. A.: Cystitis, prevention of 105
Kissinger, John B.: Submission to bite of yellow fever mosquito.... 624

LARYNX, tuberculosis of, symptomatology of ............... 435

Leprosy bacillus, success in cultivating ........................... 623

Lewis: Uro-genital tuberculosis ... 643

**Literary Notes:**

American journal of surgery... 468
English-Chinese lexicon on medical terms, Dr. Cousland. 293
Journal of Pharmacology and Experimental Therapeutics .. 642
Lee & Febiger—announcement. 62
Light therapeutics, Modern Medicine Publishing Co. .... 641
Philadelphia Academy of Surgery—announcement .......... 119
Philosophy of long life, John Lane Company ............. 641
Saunders, W. B. Co.—descriptive catalogue .............. 468
William Wood & Co.—announcement ............... 293

Lobes, prostatic, presentation of specimens ..................... 666
Loveland: Guess-work in the practice of medicine................. 656
Lumbago of febrile affections, urotropin in ...................... 154
Lumbar anesthesia, results of in gynecological operations ........ 323

MAXWELL: Adenoids in children baneful ................ 273
McCosh and Bull, Honoring ...... 507
Medical College, one for Louisville 171
Examiners, State Board of 275
Profession. new duty of... 314
Society of State of New York, committee appointed ............... 509
course. improved ......... 527
education, conference on .. 566
Medicine preventive. at Panama .. 69
Buffalo Academy of, activities and interests of .................. 83
sociological ............ 179
scientific, education of people in ............. 314
preventive. some essentials of .............. 371
, guess-work in practice of 656
Medico-legal ........................ 96
Medinal-Schering. experiences with 494
Meningitis. therapeutic value of urotropin in ..................... 616

Page

Messina earthquake, description of.. 526
Milk cans paper, in use in England 54
 pure, crusade of Chicago health department ......... 450
Military surgeon, the ............. 505
Miller, D. J. M.: Note on poisoning by egg ..................... 491
Mineral nutrients, air, water and salts ......................... 295

**Miscellany:**

Army Medical Corps Examinations ........................ 584
Bureau of Public Health and Marine Hospital Service..... 351
Medical Interne—Government hospital for insane .......... 584
Public health and marine hospital service .................. 642
State civil service commission examinations ............... 178
Surgeon General of Army—announcement by ............. 294
United States civil service commission examination. 178, 352, 526
United States Civil Service Commission ................ 688

Moet & Chandon White Seal Champagne ......................... 344
Moles, tubal ...................... 379
Mosquitoes, warning against....... 679
Municipal contagious disease hospitals ......................... 195
Munson, Edward: The citizen doctor .............................. 477

NATIONAL volunteer emergency service reorganised ..... 53
Navy given its woman nurse corps. 53
 to have medical reserves ..... 511
Neisser: Arsacetin in treatment of syphilis ......................... 537
Nephritis scarlatinal, urotropin in.. 446
New England: Tercentennial exposition in Boston in 1920......... 625
New York Post-Graduate Medical School and Hospital ............ 109
Nutrients, mineral, air, water and salts ......................... 295

OBITUARY:

Anderson, Turner ............ 230
Ballou, Edward H...............56
Bartlett, Cornelius H.......... 401
Barnes, Lewis ................ 55
Bigelow, Alfred J............. 452
Brasted, Charles M........... 452
Bull, William Tillinghast...... 452
Carlisle, Eber S.............. 346
Day, Alfred .................. 513

Page

Edebohls, George Michael ..... 112
Eisbein, David C.............. 346
Ellinwood, Albert Groves ..... 571
Fowler, Joseph ............... 401
Garvey, James Monroe ....... 452
Gillette, Walter Robarts ....... 278
Green, Robert W............. 277
Hall, Orrin I.................. 55
Harrington, Charles .......... 229
Herschler, Albert Andrew ..... 230
Hyser, William .............. 572
Jayne, Asa W................ 277
Miller, Oscar Fitzerland ...... 230
Nicoll, Henry Denton ......... 278
Nisbet, William .............. 512
O'Neill, James M............. 55
Pettit, Alonzo ................ 278
Reamy, Thaddeus Asbury .... 513
Rich, Elton S................ 230
Scott, Addison W............. 277
Wey, Hamilton D............. 512
Wheeler, John I.............. 346
Wilmot, Allen ............... 570

Obstetric surgery ................. 18
Obstetrics, cervix in, artificial dilatation of ........... 247
 surgical idea in ........ 430
Ophthalmo-tuberculin reaction ..... 559
Ordinance against spitting in public places ..................625, 626

PANCREATIC affections, diagnosis of ..................... 473
Park, Roswell, anniversary dinner 340
 On various uses of collargol 495
Patient's point of view, from the... 469
Pedersen: Acute gonococcic urethritis .............................. 546
Pertussis, pyrenol in, and asthma... 103

**Personal:**

Borzilleri, Charles R.; Dugan, John; Walker, J. E. ........ 54
Brush, Edward N.; Benedict, A. L.; Bennett, Arthur G.; Wilcox, Dewitt G.; Ross, Renwick R. ............... 345
Carroll, Jane Wall; Rogers, Howard J.; Leo-Wolf, Carl G.; Dorr, L. Bradley; Tompkins, Carl S.; Gallagher, James L. ................... 112
Cott, George F.; Colwell, Walter A.; Krauss, William C.; Brennan, Joseph P. ......... 684
Erdmann, John F.; Gould, George M.; Ruf, Frank A. ...229
Koehler, Frederick W.; Hitzel, Gustav A.; Pulver, Arthur Le Roy; Cott, George F. ... 511
Krauss, William C.; Lewis, F. Park; Wende, Ernest ...... 401
Mathews, Joseph M. ......... 450

Page

McMurtry, Lewis S.; Kenerson, Vertner ................ 626
Park, Roswell; Trick, Harry R.; King, James E.; Jack, George N.; Howe, Lucien; Allen, W. L. ................ 571
Park, Roswell; Cott, George F. ........................ 276
Peterson, Frederick; Osborne, Thomas B.; Taylor, William G.; Grant, John H.; Brown, Clayton M.; Gorgas, Col. William C.; Park, Roswell; McMurtry, Lewis S......... 683
Pohlman, G. Augustus; Carroll, Jane Wall; Brecht, F. E. L.; Kirchner, Walter C. G.;
Reed, Charles A. L.; MacDonald, Carlos; Brush, Frederic; Dr. and Mrs. D. C. Eisben... 277
Spratling, William P.; Cott, George F. .................. 55
Stockton, Charles G. .......... 400
Tompkins. Carl S.; Breuer, Max; Wilcox, Dewitt G. .... 173
Van Peyma, P. W.; Stoddart, James; Smith, Dr. and Mrs.. Edward T.; Drs. Park and McGuire .................... 627
Wagner. George G.; Hurley, Patrick J.; Porter. Eugene H.; Goldberg, Jacob; Smith, Alexander; Frost. Edward L.; Davidson, William R.; Bowerman, E. A. .......... 451

Physicians, institutional work for young .......................... 565
Plehn: Collargol in joint rheumatism ............................ 496
Pneumonia croupus, pyrenol in..... 103
Pneumopericardium, report of case. 531
Porter: Prostatectomy, early ...... 104
Prato: New hypnotic medinal in psychiatric practice ............. 493
Pregnancy extrauterine, experiences in .............................. 237
Proteid iron preparations.......... 32
Public places, ordinance against spitting in ..................625, 626
Puerperal eclampsia. renal decapsulation for ............ 305
endometritis. treatment of 331

RACHFORD: Collargol to children ......................... 496
Railway wrecks, causes of ........ 275

SCARLET fever, treatment of.... 495
Scientific medicine, education of people in ...................... 314
Schieffelin: Suggestion with reference to a prohibitory duty on cocaine ........................ 682
Schurman, Jacob Gould: Opposition to antivivisection bills ........... 622
Seidel, Curt: Collargol enemata in septic affections ................. 155
Seminales, vesiculæ, chronic inflammation of ...................... 28
Shattuck, F. C.: Typhoid, urotropin in convalescence from .......... 103
Sheffield, H. B.; Chronic constipation in children ................. 102
Smallpox, vaccination for ......... 311

**Society Meetings:**

American Association of Obstetricians and Gynecologists ........................56, 110, 174
American Dermatological Association ........................ 174
American Medical Editors Association .................... 630
American Medical Association ........................ 513, 629
American Protologic Society... 629
Association of Erie Railway Surgeons ................... 174
Association of American Medical Colleges .............453, 514
Buffalo Academy of Medicine ...231, 279, 347, 453, 515, 669, 627
Buffalo Medical Union ........ 453
Clinical Club .................. 453
International Congress on Tuberculosis ............113, 116
Medical Association of Central New York ..........173, 230, 278
Medical Society of State of New York, Eighth District Branch 175
Medical Society of the County of Erie ....230, 346, 453, 608, 684
Medical Society of State of New York ...................... 346
Medical Society of County of Cattaraugus ................ 515
Medical Society of County of Niagara .................. 629
Mississippi Valley Medical Association .................116, 231
National Confederation of State Medical Examining and Licensing Boards ............ 630
Rochester Academy of Medicine ...........280, 347, 455, 516, 628
Roswell Park Medical Club.... 453
Southern Surgical and Gynecological Association .......... 230

**Society Proceedings:**

Buffalo Academy of Medicine .......................43, 85, 210
Department of Health ......... 220
Esculapian Club .............. 217
International Congress of Tuberculosis ...................... 254

Page

Medical Society of the County of Erie ......40, 216, 251, 381, 492

Sociological medicine ............. 179
Spratling: Institutional work for young physicians ................ 565
State Education Building.......... 618
Health Department: Amendment in reference to birth and death certificates...... 681
Stenitz, Ernst: Veronal group, soluble hypnotic of ................ 157
Stern, Richard: Urine, influence of urotropin on .................... 153
Stocker: case of erysipelas......... 667
Stomach and bowel disturbances, overlooked causes for ........... 142
Surgery, obstetric ................. 18
next twenty-five years in. 585
Surgeon, the military ............. 505
Surgeons, International Congress of 595
Surgical idea in obstetrics ......... 430
Syphilis, employment of arsacetin in treatment of ................ 536

TACHYCARDIA, treatment of.. 485
Therapeutic notes ........... 99

**Topics of Public Interest:**

At last, the oxygen bracer...... 159
Antituberculosis crusade at the county fairs ................ 105
Committee to fight tuberculosis in Buffalo .................. 394
Fight against consumption .... 336
Infection, how it affects the body 436
Medicolegal .................. 266
Milk, pasteurisation of ........ 165
New York Police to stop noises 47
Nutritive and medicinal value of alcohol .................. 561
State Board of Medical Examiners hold court ......... 332
State Charities Aid Association of New York .......... 500
Smoking, chemistry of ........ 497
Unclean and dangerous habit... 499
Vanderbilt tenements ......... 501
Wiley, H. W., visits Buffalo... 335

Tubercle bacilli, staining of ........ 67
Tuberculosis, International Congress on ............... 51
Milk Exhibit at International Congress of 110
Cuguillere's Serum in treatment of ...... 156
exhibit at Washington 169
strong fight against inroads of ........ 343
problem in Buffalo .. 411
of larynx, symptomatology of ........ 435
fight against ........ 625
uro-genital .......... 643
Typhoid, urotropin in convalescence from .................. 103
Fever, new dietetic and injection method of treating ................ 222
fever, diet in ............ 361

UNIVERSITY day ............ 508
of Buffalo 1846-1909....675
of State of New York, appointments made at meeting of .......... 680
United States Government: representation of at five medical conferences in Europe ............. 681
Urethritis, acute gonococcic, treatment of ........................ 546
Urine, influence of urotropin on.... 153
retention of and treatment.. 557
Urotropin hexamethylenamin, excretion of pancreatic juice ................. 44
in convalescence from typhoid .............. 103
in scarlatinal nephritis... 446
in lumbago of febrile affections ............. 154
Urotropin, excretion of, in cerebrospinal fluid ..................... 616
Uterus, retroversions of ........... 421

VACCINATION for smallpox... 311
Vagina, double ............. 556
Valeric, a new, brovalol ........... 331
Veronal group, soluble hypnotic of. 157
Veins varicose, surgical treatment of 63
Vesiculæ seminales, chronic inflammation of ..................... 28

WEBSTER, William M.: Why is doctor on same plane with a dog ..................... 622

Weitlaner, Franz: Urotropin in lumbago of febrile affections..... 154

Whooping cough, treatment of...... 95

ZWEIG: Hemorrhoids .......... 102

